# EVmiRED: a curated database of miRNA editing landscape in extracellular vesicles

**DOI:** 10.1007/s44307-026-00107-w

**Published:** 2026-04-09

**Authors:** KaHei Kou, Lanqi Wen, Luo Yang, Zhoufeng Gao, Bei Jin, Jingxuan Pan, Rui Zhang

**Affiliations:** 1 State Key Laboratory of Ophthalmology, Guangdong Provincial Key Laboratory of Ophthalmology and Visual Science, Zhongshan Ophthalmic Center, Sun Yat-Sen University, Guangzhou, China; 2 MOE Key Laboratory of Gene Function and Regulation, Guangdong Province Key Laboratory of Pharmaceutical Functional Genes, State Key Laboratory of Biocontrol, School of Life Sciences, Sun Yat-Sen University, Guangzhou, China

**Keywords:** Database, miRNA editing, Extracellular vesicles

## Abstract

Metastasis accounts for the vast majority of tumor-related mortality. Certain populations of tumor cells exhibit organotropism by preferentially colonizing specific distant organs. The organ specificity of metastatic cells is determined by unique interactions between tumor cells and the microenvironment in target organs. Tumor extracellular vesicles (EVs), particularly exosomes, delivering tumor cell components including nucleic acid complexes, proteins, and lipids, play a crucial role in mediating intercellular communication between tumor cells and their microenvironment. ADAR-mediated microRNA (miRNA) editing has emerged as a crucial mechanism influencing miRNA stability, processing, and target specificity. Although EVs are increasingly recognized as important vehicles of intercellular signaling and promising biomarkers for cancer, the landscape of miRNA editing within EVs remains largely unexplored. Here, we present EVmiRED (Extracellular Vesicle miRNA Editing Database), a resource that integrates miRNA expression and editing profiles from tumor-derived EVs. The current release includes data from 683 samples across 12 tumor types and cell lines. EVmiRED provides detailed information on miRNA abundance, editing frequency, and the predicted functional impact of specific editing events. EVmiRED enables users to query individual miRNAs, visualize expression and editing patterns, and access raw datasets for customized analyses. Together, EVmiRED offers a valuable platform to advance our understanding of RNA editing-mediated regulation in intercellular communication, tumor progression, and cancer immunology.

## Introduction

Metastasis, the development of secondary tumors in distant tissues, accounts for the vast majority of tumor-related mortality. Tumor cells often display striking organotropism, preferentially colonizing specific sites such as the liver, lung, bone, and brain (Valastyan and Weinberg 2011; Dunbar et al. 2025). Successful metastatic colonization requires tumor cells to overcome the unique and often hostile microenvironments of distant tissues. This process critically depends on dynamic and reciprocal interactions between disseminated tumor cells and resident host cells within the target organ (Peinado et al. 2017; Gan et al. 2024). Such communication occurs through soluble factors, direct cell–cell contact, or EVs.

Tumor-derived EVs play central roles in cancer progression, metastasis, therapeutic resistance, and immune modulation (Kalluri and LeBleu 2020; Greening et al. 2025; Bayat and Sadri Nahand 2024). Among EV subtypes, exosomes are small vesicles with an average diameter of approximately 100 nm (Kalluri and LeBleu 2020). They transport diverse bioactive molecules, including nucleic acids, proteins, lipids, and metabolites, to recipient cells both locally and systemically, thereby promoting the metastatic niche formation (Greening et al. 2025; Liu et al. 2021). For example, tumor-derived exosomal integrins determine organ-specific metastatic tropism (Hoshino et al. 2015).

Among the various molecular components of EVs, miRNAs are small RNA molecules that regulate gene expression by binding to mRNAs, either degrading them or preventing their translation. They are involved in important biological processes and disruption in their function can lead to diseases like cancer (Shang et al. 2023). For example, breast cancer-derived exosomal miR-200 can reprogram non-metastatic cells into a metastatic phenotype (Le et al. 2014).

Recent advances in high-throughput sequencing technologies have facilitated the discovery of miRNA alterations in various cancers, such as breast, lung, colorectal, and prostate cancers (Croce 2009; Lu et al. 2005). These studies have underscored the potential of miRNAs as non-invasive biomarkers for early cancer detection and as indicators of treatment response. However, while miRNA expression profiling has been extensively studied, miRNA editing, a post-transcriptional modification that can profoundly alter miRNA processing, stability, and target recognition, remains less explored. In particular, adenosine-to-inosine (A-to-I) RNA editing catalyzed by ADAR enzymes can modify miRNAs, affecting their precursor processing by Drosha or Dicer or altering their binding specificity in the seed region (Li et al. 2018; Liao et al. 2020; Gebert and MacRae 2019; Yang et al. 2006). Notably, the overlap between the predicted mRNA targets before and after editing is only 3% on average, thereby significantly reshaping their target networks (Alon et al. 2012). Dysregulated miRNA editing has been increasingly linked to cancer development and metastasis (Liao et al. 2020; Wang et al. 2017).

**Fig. 1:**
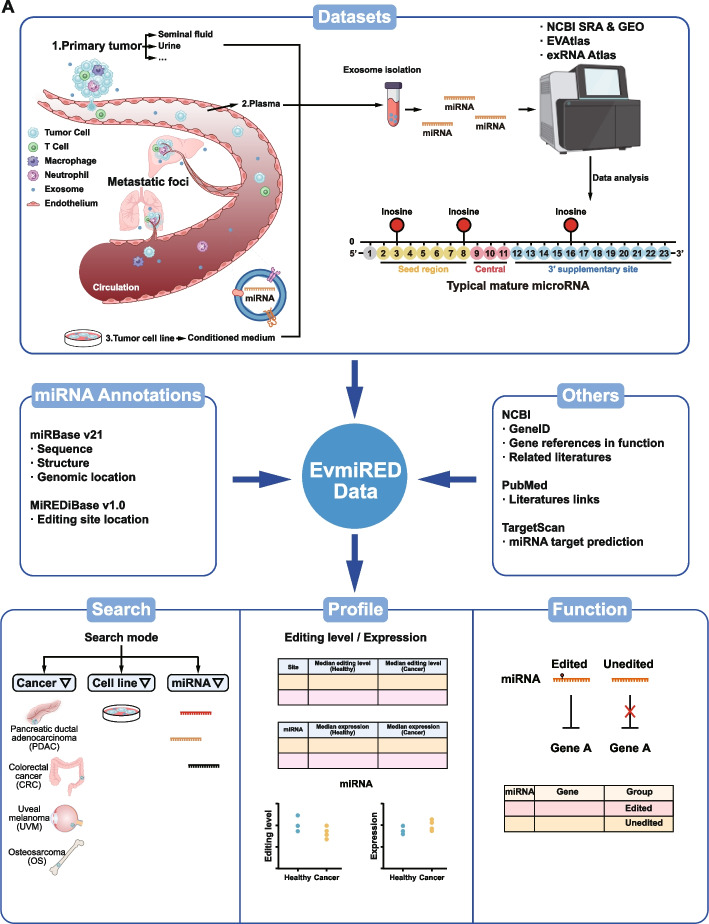
Overview of the EvmiRED. EvmiRED platform focuses on microRNA editing and expression in cancer. It integrates miRNA annotations and offers functionalities such as searching, profiling, and exploring miRNA functions, linking to related literature and pathways

Despite growing interest, systematic studies of miRNA A-to-I editing in EVs are still scarce. Existing databases, such as Vesiclepedia, ExoCarta, and EVpedia (Kalra et al. 2012; Keerthikumar et al. 2016; Kim et al. 2013), provide valuable catalogs of EV contents but lack detailed annotations of miRNA expression and editing. Similarly, resources like EVmiRNA (Liu et al. 2019) and EVAtlas (Liu et al. 2022) focus primarily on miRNA abundance, without incorporating information on editing events. To bridge this gap, we developed EVmiRED (https://evmired.sysu.edu.cn/), a comprehensive database that integrates both miRNA expression and editing profiles in EVs derived from tumor and normal samples (Fig. 1). EVmiRED represents the first resource dedicated to the systematic characterization of miRNA editing within EVs. By enabling exploration, comparison, and visualization of these features across cancer types, EVmiRED provides a valuable platform for identifying novel biomarkers, elucidating miRNA editing dynamics, and advancing the development of liquid biopsy tools for cancer diagnosis.

## Materials and methods

### Small RNA-seq data collection

We first explored the EVmiRNA and exRNA Atlas databases to construct the initial EVmiRED datasets, and subsequently searched small RNA-seq data in the NCBI Sequence Read Archive (SRA) using specific keywords such as “extracellular vesicles,” “exosomes,” “microvesicles,” and “*Homo sapiens*” [orgn], restricting the search period to 2015–2025. The retrieved datasets were manually filtered, and those lacking information on exosomes or miRNAs were discarded. In addition, studies focusing on non-cancerous lesions or non-solid tumors were excluded from the analysis.

For the collected datasets, we first categorized them according to cancer type (e.g., breast, colon, gastric, liver, and kidney cancers). We also recorded their corresponding source publications, sample origins (predominantly plasma, serum, and cell lines, with a minority from urine, tumor tissue, etc.), and sequencing methodologies (e.g., single-end or paired-end sequencing of miRNAs or ncRNAs).

Following the guidelines of the International Society for Extracellular Vesicles (ISEV), we applied stringent sample collection and isolation criteria to classify each dataset as either exosome-derived or microvesicle-derived. Datasets with ambiguous experimental descriptions or insufficient raw reads were removed. For the remaining datasets, we performed additional quality filtering by discarding reads that (i) contained more than five ambiguous bases (N), (ii) exhibited abnormal lengths after adapter trimming (< 15 nt or > 25 nt), or (iii) had an average Phred quality score ≤ 20. Finally, any dataset retaining fewer than one million clean reads after filtering was excluded from further analysis.

### Identification of miRNA editing sites from small RNA-seq data

We first constructed an unedited pre-miRNA database using the reference sequences of pre-miRNAs without any modifications, and custom edited pre-miRNA databases by replacing A with G at known editing positions within the mature miRNA region. These positions were identified in our previous studies using miR-mmPCR, a method that allows for the detection of specific miRNA editing events (Li et al. 2018).

Small RNA-seq data were obtained from the NCBI SRA (https://www.ncbi.nlm.nih.gov/sra/). Low-quality reads were removed, and adapter sequences were trimmed using Cutadapt with parameters –q 20,20 --trim-n. Identical reads were collapsed, and only reads with lengths between 16 and 25 bp were retained. Because the 3′ end of animal miRNA sequences is often subject to A or U addition, the last two bases of a read were trimmed if they were A or U (Burroughs et al. 2010). 

The cleaned reads were first aligned by BLAST against the unedited pre-miRNA database (E-value < 0.1; no mismatch allowed) (Altschul et al. 1990). Unmapped reads were then aligned by BLAST against the custom edited pre-miRNA database under the same parameters. This two-step alignment approach ensured the comprehensive identification of both unedited and edited miRNA sequences. To ensure accuracy, we selected datasets with ≥ 60% of bases retained after trimming and with ≥ 0.1 million reads (16–25 bp) mapped to the miRNAs. Editing positions were identified by filtering out low-quality Gs (base quality score < 30) and requiring: (i) support from ≥ 2 editing reads, (ii) ≥ 5% editing levels, and (iii) statistically significant modification (Benjamini–Hochberg adjusted *p* < 0.05) relative to the sequencing error rate (Alon et al. 2012). Finally, putative edited miRNAs were mapped to the reference genome, and sequences that perfectly matched the genome were excluded to ensure specificity.

Editing levels were calculated as the ratio of mismatched bases to total coverage at each position, expressed as follows: Editing level = (mismatch count)/(coverage depth). High-confidence editing sites in expressed EV miRNAs were defined by stringent criteria: a minimum coverage of at least two reads and detection in at least ten samples.

Expression levels were given in reads per million (RPM) and were retained only if ≥ 10 RPM. RPM was calculated as follows: (miRNA mapped reads/total mapped reads) × 10⁶.

To mitigate heterogeneity arising from batch effects and differences among technical platforms, batch correction of miRNA expression was performed using pyComBat (Behdenna et al. 2023). Both the uncorrected (raw) and batch-corrected normalized expression values are provided in the website for downstream analyses.

### Integration of miRNA profiling and annotation

Basic annotations for all mature miRNAs were retrieved from miRBase v21 (Kozomara et al. 2019). Functional descriptions, literature links, and editing site information were collected from NCBI GeneRIF, PubMed, and MiREDiBase (Marceca et al. 2021). For each cancer type or condition, both the editing level and expression abundance of every miRNA were reported as the mean values across all included projects.

### PCA analysis

Because the number of editing sites was limited, we did not exclude sites with missing editing values in more than one-third of samples. Instead, missing values within each condition were imputed using a DecisionTreeRegressor from the Python scikit-learn package with default parameters. All editing sites were included in the PCA if their comparison between the healthy group and at least one cancer type yielded a *p*-value < 0.1.

### miRNA target prediction

Putative targets of the unedited and edited miRNAs were predicted with TargetScan7 (Agarwal et al. 2015). The program was run separately on the unedited miRNA sequence and on the edited sequence in which each inosine was converted to guanosine, because I:C and G:C base pairs contribute similarly to target hybridization (Agarwal et al. 2015). The putative targets of canonical 7–8 mer 3′ UTR sites were considered. All human 3′ UTR sequences were downloaded from the TargetScan portal (http://www.targetscan.org/).

### Database and website implementation

The EVmiRED web portal was built using Python FastAPI, which powers the RESTful API layer.

## Results

### miRNA editing data processing

Although mature miRNAs make up the majority of EV miRNAs, detecting mature RNA-editing events within them using small RNA-seq is inherently challenging due to the short reads (around 22 nt), which often map poorly to the reference genome when carrying mismatches (Alon et al. 2012; de Hoon et al. 2010). To overcome this limitation, we leveraged the high-confidence editing sites previously identified by our miR-mmPCR-seq (Li et al. 2018) method to construct a curated reference database of edited pre-miRNAs, which served as the foundation for downstream analyses.

**Fig. 2:**
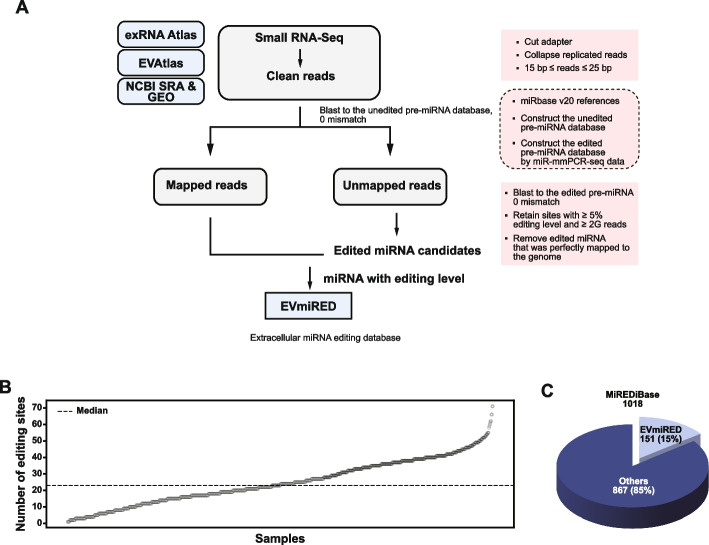
Workflow and outcomes of the miRNA editing analysis pipeline. **A** Schematic representation of the pipeline used to identify edited miRNAs from small RNA-Seq data. **B** Distribution of edited sites across samples (N = 683). **C** Pie chart showing the proportion of edited miRNAs in the EVmiRED database relative to the total number of miRNAs in the MiREDiBase database

By analyzing 683 samples, including tissues and cell lines (Fig. 2A), we detected a median of 22 miRNA editing sites per sample (Fig. 2B). Previously, only 11 editing sites have been reported in EV miRNAs (Nigita et al. 2018; Shelton et al. 2018). In contrast, our datasets identified 151 edited sites, representing approximately 15% of all known editing events reported in MiREDiBase (Marceca et al. 2021), revealing that EV miRNA editing is far more abundant than previously appreciated (Fig. 2C).

### Editing event distribution in EV miRNAs

**Fig. 3:**
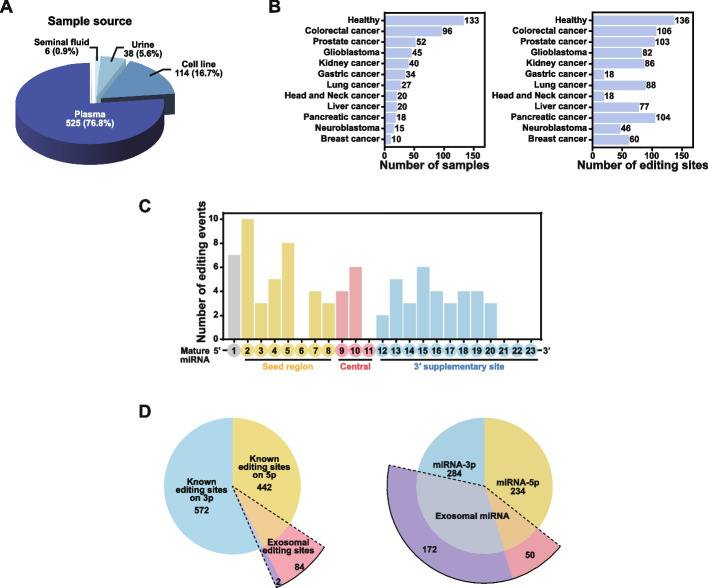
Characterization of EV miRNA expression and editing. **A** The sample sources of small RNA-seq data. **B** Bar plots display the number of samples and editing sites across various cancer types and healthy controls derived from plasma samples. **C** The distribution of editing sites on the mature miRNAs in plasma. **D** Left: Pie chart illustrates the proportion of edited sites in plasma samples relative to the total number of sites in the MiREDiBase database. Right: Pie chart shows the expression proportion of mature miRNA strand

In the current version of our database, we compiled a comprehensive collection of human small RNA-seq datasets primarily derived from various biofluids and cell lines (Fig. 3A). The database encompasses a total of 683 curated samples incorporated into our system. Notably, plasma-derived samples accounted for 76.9% of the datasets, underscoring the predominant use of plasma in EV research and providing a robust foundation for investigating miRNA editing events and their biological implications (Fig. 3A). In the plasma-derived samples, the healthy control group (n = 133) and the remaining 392 samples span multiple cancer types. The number of detected sites in each group is influenced by factors such as sequencing depth across datasets, EV extraction protocols, and EV subtype composition. Therefore, both biological signals and technical heterogeneity must be considered, and differences in the number of editing sites cannot be attributed solely to health status or cancer type (Fig. 3B).

Within the plasma datasets, we identified 86 high-confidence editing sites. Among these, editing events were most frequently observed at positions 2 and 5, both of which fall within the seed region of miRNAs (Fig. 3C). Given the critical role of the seed region in target recognition and binding, frequent editing at these positions suggests a potential modulatory impact on miRNA-mRNA interactions, thereby influencing downstream gene regulation.

Strikingly, 84 out of 86 editing sites were located on the 5′ strand of miRNAs (Fig. 3D). Although 5p miRNAs constitute only 29% of the expressed high-confidence EV miRNAs, they harbor the vast majority of editing events, indicating that RNA editing in EV-derived miRNAs predominantly occurs on the 5′ strand rather than the 3′ strand. In contrast, our previous study (Li et al. 2018) reported no significant difference in editing site distribution between the 3′ and 5′ arms of cellular miRNAs. This discrepancy suggests the involvement of additional regulatory mechanisms governing strand-specific editing in EV miRNAs.

In summary, the identification of high-confidence editing sites, particularly those within the seed regions, highlights their potential to reshape EV miRNA target specificity and thereby influence diverse biological processes and disease states.

### Case study: condition-specific variability in miRNA editing profiles

**Fig. 4:**
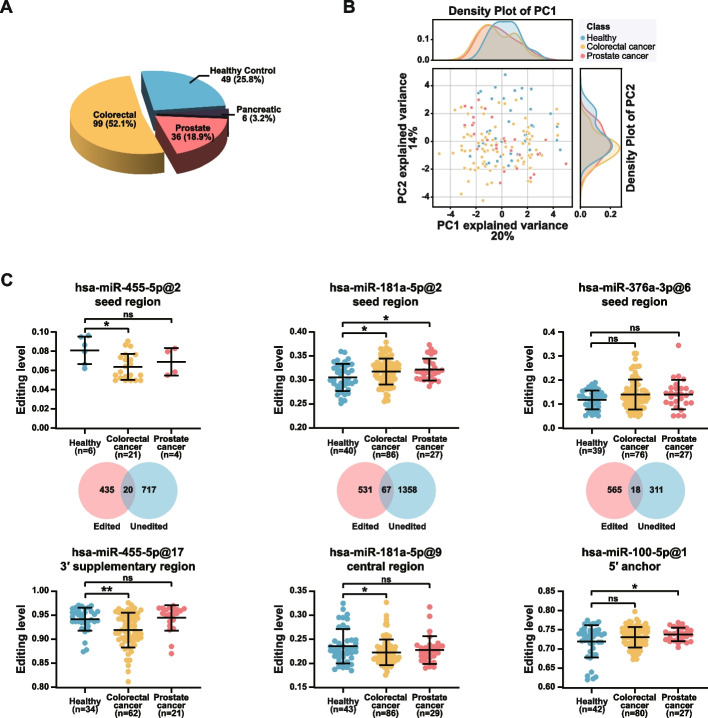
Analysis of miRNA editing patterns across different cancer types and healthy controls in the case study. **A** The distribution of samples categorized by disease status provides a comprehensive overview of the datasets. **B** The Principal Component Analysis (PCA) of miRNA editing profiles, illustrating the separation between healthy controls and cancer patients. **C** Boxplots depicting differential editing levels of specific miRNAs in healthy individuals and patients with colorectal and prostate cancers. The miRNA sequence is separated into five functional domains that affect miRNA target recognition: 5′ anchor (nt 1), seed sequence (nts 2–8), central region (nts 9–12), 3′ supplementary region (nts 13–16), and 3′ tail (nts 17–22). For editing sites located within the seed region, the overlap of target genes between unedited and edited miRNAs is also shown. *p < 0.05, **p < 0.01

To demonstrate the utility of the database, we selected a single dataset for a case study. Specifically, we analyzed miRNA editing levels across multiple conditions and miRNA types using data from Tiezheng Yuan’s 2016 study (Yuan et al. 2016) (Fig. 4A). The PCA analysis revealed distinct separations among conditions, indicating substantial variability in miRNA editing patterns (Fig. 4B). This condition-specific variability suggests that miRNA editing is dynamically regulated and may play context-dependent functional roles.

Further analysis of the PCA-selected miRNAs that most strongly distinguished healthy individuals from patients with colorectal or prostate cancer revealed significant differences in editing levels (Fig. 4C). Several editing events occurring in distinct regions, including the seed region, 3′ supplementary site, and 5′ supplementary site, showed significant differences between healthy and diseased groups. For example, position 2 of hsa-miR-181a-5p and hsa-miR-455-5p showed significant increases and decreases, respectively, in seed-region editing in colorectal cancer compared with healthy controls. Conversely, position 1 of hsa-miR-100-5p, located in the 5′ anchor region, exhibited an increase in editing in prostate cancer.

Target prediction using TargetScan (Agarwal et al. 2015) further indicated that editing variations within the seed region may substantially alter miRNA target recognition (Fig. 4C), potentially leading to extensive target network rewiring.

Collectively, these findings highlight the condition-specific nature of EV miRNA editing and underscore its potential as a biomarker for cancer diagnosis and as a modulator of post-transcriptional gene regulation.

### User interface and data accessibility

**Fig. 5:**
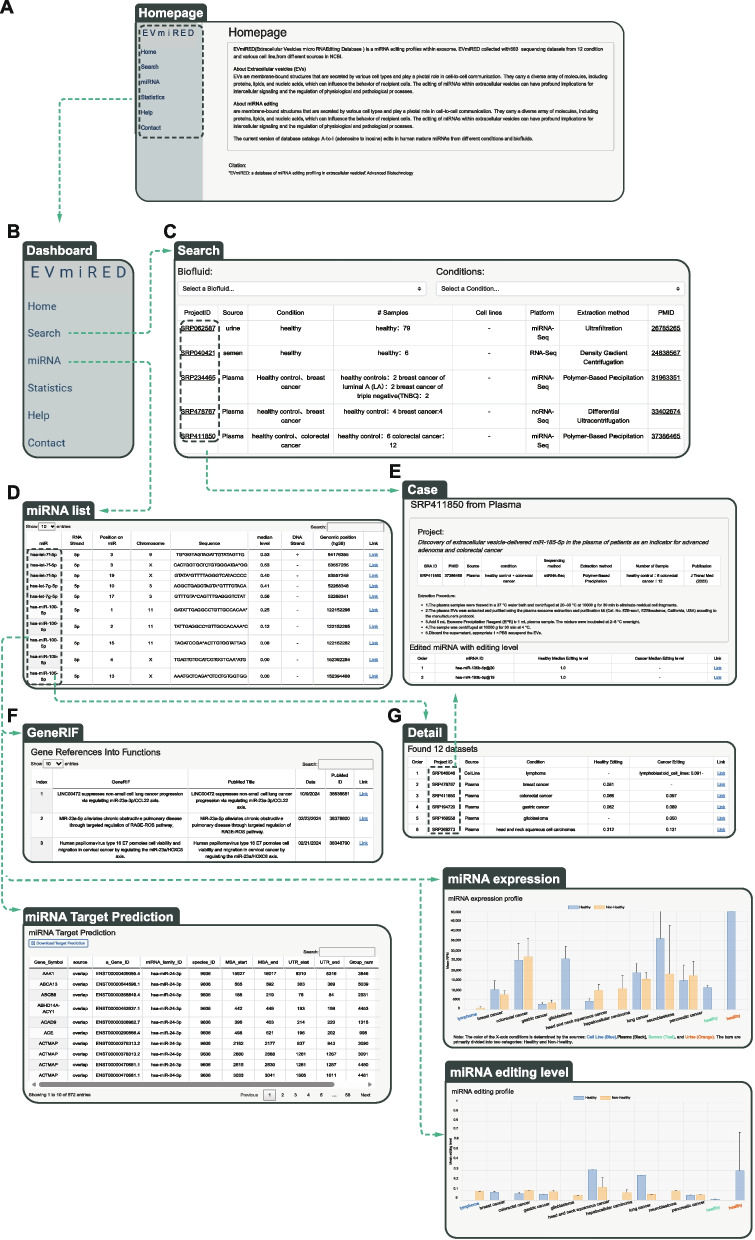
Overview of the web interface and its functionalities. **A** The homepage serves as the entry point, providing users with an overview and easy access to the main features. **B** The dashboard control enables users to navigate between different pages, enhancing the user experience. **C** The search functionality based on cancer type and condition allows users to filter and retrieve data relevant to specific cancers and associated conditions. **D** The Search allows users to search for certain miRNAs on the miRNA list. **E** The case page allows users to access detailed editing profiles and expression profiles for specific datasets. **F** The interface allows users to search for the functions of miRNAs providing valuable insights into their roles. **G** The interface reports which datasets include a given miRNA list and provides one-click navigation to the corresponding projects. **H** The interface displays the predicted mRNA targets of both the unedited and edited forms of a miRNA, allowing users to compare how their target spectra differ. **I**-**J** After identifying the intersecting datasets, the browser simultaneously displays the editing and expression profiles of miRNA for direct comparison

EVmiRED provides an intuitive and user-friendly web interface that allows users to access and analyze data without requiring prior bioinformatics expertise (Fig. 5). The database offers two main entry points for exploration: the Search module and the miRNA module. Each module begins with a modal box that enables filtering of miRNA editing sites based on specific conditions.

The Search module offers four filtering parameters: biofluid source (e.g., plasma), genomic region (e.g., chromosome or miRNA), and, optionally, biological source (e.g., breast cancer). Upon query submission, the module generates a table listing the identified editing sites, accompanied by essential metadata based on the selected filters. This includes miRNA name, modification type, chromosome, strand, genomic position, relative positions within pre-miRNA and mature miRNA, detection strategies used, and the mature miRNA sequence.

The module also provides the expression levels of miRNAs associated with these editing sites, enabling users to evaluate the potential functional impact of each event. Additional information, such as publications, external resources, biological sources, sequencing strategies, and functional annotations, can be accessed via interactive buttons, facilitating rapid interpretation and hypothesis generation regarding the biological relevance of miRNA editing events.

Results from each module can be conveniently downloaded using the provided buttons. The Compare module enables users to examine editing and expression levels of specific miRNAs across different datasets or disease conditions, facilitating comparative and integrative analyses.

Additionally, users can directly download miRNA editing and expression datasets under various conditions through the Download module, supporting in-depth analysis and downstream research applications.

## Discussion

The establishment of an EV miRNA editing database provides a valuable resource for decoding the complex intercellular communication networks mediated by non-coding RNAs. Unlike conventional repositories, this platform integrates multidimensional datasets into a unified analytical framework, encompassing cancer-type-specific expression profiles (e.g., breast, colon, and gastric carcinomas) as well as diverse biofluid sources, including plasma-, serum-, and tissue-derived EVs.

Our current analysis adopts a conservative strategy by anchoring EV miRNA editing detection to previously reported editing sites in pre-miRNAs. While this approach enhances confidence in identified events, it likely represents a lower bound of the true extent of miRNA editing within EVs. Future iterations of the database will expand beyond known pre-miRNA editing sites by generating matched pre-miRNA and EV-derived small RNA sequencing datasets, enabling systematic discovery of novel EV editing events.

Integrating and visualizing miRNA expression and editing profiles within EVs offers powerful opportunities for cancer research. Such datasets can accelerate the discovery of novel biomarkers and facilitate the development of non-invasive diagnostic tools. Ultimately, this line of investigation holds great promise for advancing early cancer detection, monitoring therapeutic responses, and enabling personalized treatment strategies.

Moreover, EVmiRED provides a foundation for identifying miRNA editing events that may influence tumor progression, metastasis, or therapeutic resistance. Exploring the role of ADAR enzymes in shaping the EV miRNA landscape could reveal new prognostic biomarkers and therapeutic targets. In addition, elucidating how RNA editing is regulated within EVs will deepen our understanding of RNA-based intercellular communication and shed light on the molecular mechanisms underlying human diseases such as cancer.

To further extend and continuously update this resource, future efforts will focus on expanding the cancer spectrum to include distinct histological and molecular subtypes; incorporating early- versus advanced-stage comparisons, initially emphasizing colorectal and pancreatic cancers; and integrating longitudinal samples collected before, during, and after therapy to capture the dynamic evolution of EV miRNA editing. Together, these developments will strengthen the clinical relevance of EV-associated RNA editing and establish EVmiRED as a continually evolving data hub for translational oncology.

## Data Availability

https://evmired.sysu.edu.cn/.
